# Decisions about prophylactic gynecologic surgery: a qualitative study of the experience of female Lynch syndrome mutation carriers

**DOI:** 10.1186/s13053-015-0031-4

**Published:** 2015-03-19

**Authors:** Holly Etchegary, Elizabeth Dicks, Kathy Watkins, Sabrina Alani, Lesa Dawson

**Affiliations:** 1 Division of Medicine, Clinical Epidemiology Unit, Memorial University Health Sciences Centre, Patient Research Centre, Room 1414 300 Prince Phillip Drive, St. John’s, A1B 3V6 NL Canada; 2 Center for Health Informatics & Analytics (CHIA) Health Sciences Centre, Room 1756 300 Prince Philip Drive, St. John’s, A1B 3V6 NL Canada; 3 Centre for Nursing Studies, Southcott Hall, 100 Forest Road, St. John’s, A1A 1E5 NL Canada; 4 Department of Oncology, Memorial University, St. John’s, A1B 3V6 NL Canada; 5 Obstetrics and Gynecology Division of Gynecologic Oncology, Memorial University, 300 Prince Phillip Drive, St. John’s, A1B 3V6 NL Canada

**Keywords:** Lynch syndrome, HNPCC, Prophylactic surgery, Gynecological

## Abstract

**Background:**

Women who carry a mutation for Lynch syndrome face complex decisions regarding strategies for managing their increased cancer risks. At present, there is limited understanding of the factors influencing women’s prophylactic surgery decisions.

**Methods:**

As part of an exploratory pilot project, semi-structured interviews were conducted with 10 women who were Lynch syndrome mutation carriers and had made prophylactic surgery decisions. Nine of 10 women had chosen to undergo prophylactic hysterectomy and/or oophorectomy as a means of managing their increased gynecological cancer risks.

**Results:**

Study findings revealed that surgery decisions were influenced by multiple factors, including demographic variables such as age and parity, as well as psychosocial factors such as cancer worry, in addition to personal and social knowledge of gynecological cancer. While all women were satisfied with their surgery decision, some reported they were not fully informed about the negative impact on their quality of life post-surgery (e.g., complications of surgically-induced menopause), nor about the potential for, or risks and benefits of, hormone replacement therapy.

**Conclusions:**

Study findings highlight some of the factors associated with prophylactic surgery decisions and women’s perceptions about pre-surgical information provision and needs. Suggestions are made for improving the information and support provided to female carriers of a Lynch syndrome mutation.

## Background

Hereditary non-polyposis colorectal cancer (HNPCC) or Lynch sndrome (LS), accounts for 3-5% of all colorectal cancers [1],[2]. It is a dominantly inherited syndrome, predisposing carriers to a high risk of early-onset colorectal cancer [1]-[5]. LS is caused by mutations in four mismatch repair genes: *MLH1, MSH2, MSH6,* and *PMS2*[1]. In addition to their increased colon cancer risk, women carrying these germline mutations have dramatically elevated rates of gynecological cancer compared to women in the general population. They face a 40-60% lifetime risk of endometrial cancer and a 10-12% lifetime risk of ovarian cancer, compared to the general population risks of ~3% and ~1.4%, respectively [1]-[3].

Currently, women at increased hereditary risk for gynecologic cancers have two risk management options: 1) increased cancer surveillance, or 2) surgical removal of the uterus (hysterectomy) and/or removal of the ovaries and the fallopian tubes known as risk-reducing salpingo-oophorectomy (RRSO). Since some Lynch-associated cancers are diagnosed before the age of 35, some authors recommend annual surveillance in this high-risk group of mutation carriers, including transvaginal ultrasonography, tumor marker CA125 blood tests and/or endometrial biopsy [6],[7]. At present, however, the benefit of screening for gynecological cancers in Lynch mutation carriers is not supported by research evidence, nor is there consensus on the optimal screening modality or timing [8]. In our jurisdiction, we found that screening did not result in earlier gynecologic cancer detection in mutation carriers, and despite screening, two young women died from ovarian cancer [9]. In contrast, prophylactic gynecological surgery (RRSO and/or hysterectomy) significantly reduces the risk of gynecological cancers. Schmeler and colleagues [10] reported that none of 61 women with LS were diagnosed with endometrial cancer after prophylactic hysterectomy, compared to 33% of women who had not undergone surgery. Lacking evidence for the effectiveness of gynecological cancer screening, it is generally recommended that females with LS consider prophylactic hysterectomy with RRSO upon the completion of childbearing since these procedures largely prevent the development of endometrial and ovarian cancers [7]-[11].

While an extensive literature exists regarding the uptake of prophylactic gynecological surgery in women carrying hereditary breast/ovarian cancer mutations (BRCA 1 and BRCA 2), fewer studies examined the surgical decisions of the female LS population. Research with women carrying BRCA mutations reveals that demographics (e.g., older age, having children), medical factors (e.g., personal or family history of breast and ovarian cancer), and psychosocial factors (e.g., high levels of cancer worry) are positively correlated with RRSO uptake [12]-[14]. Most women are satisfied with their surgery decisions, and report lower levels of perceived cancer worry, and higher levels of control following surgery [14]. Premenopausal women reported the most negative impacts following surgery [12],[13]. These included negative physical effects, such as hot flashes, vaginal dryness, reduced sexual interest and pleasure, and pain during intercourse. The literature is mixed regarding satisfaction with information received prior to surgery: some women felt they were fully informed, while others did not have enough information to make decisions about hormone replacement therapy [13]-[15]. Overall, however, the rate of satisfaction with prophylactic gynecological surgery in women with BRCA mutations is very high [12]-[15].

Considerably fewer studies focused on prophylactic surgery decisions in female LS mutation carriers. However, research suggests that women affected by LS may not understand the complexity of the diagnosis, at least prior to genetic counseling. A survey study of 65 women reported low awareness of extra-colonic cancers and decreased use of endometrial screening before genetic counseling [16]. A small qualitative study involving women at risk for familial ovarian cancer (due to family history, not Lynch syndrome) reported gaps in knowledge about ovarian cancer screening and prophylactic oophorectomy [15]. The authors suggested such gaps raised concerns about the extent to which women were making informed cancer risk-management decisions. They identified several areas where the provision of information could be improved, including practical details about the operation itself and postoperative functioning, as well as the risks and benefits of hormone replacement therapy. Compared to women carrying BRCA mutations, the risks associated with the use of HRT (e.g., increased risk of breast and endometrial cancer) are lower for women affected with Lynch syndrome [7],[12].

A recent study reported that LS carriers were compliant with guidelines for cancer risk reduction in the year following genetic testing, with nearly 70% opting for prophylactic hysterectomy by three-year follow up [17]. Research suggests prophylactic surgery decisions are complex, driven by demographics (e.g., age, parity), medical factors (e.g., mutation status or abnormal ovarian screening results) and psychosocial factors (e.g., anxiety about cancer risk) [14]-[18]. Ovarian screen-detected abnormalities (e.g., cysts) were often sufficient motivation for women to discontinue screening and opt for surgery, as was the advice of clinicians [18]. A recent study of 74 women with LS [19] found the majority were knowledgeable about aspects of gynecological screening (e.g., age at which to begin) and had accurate perceptions of their colorectal and endometrial cancer risks. However, half reported that their care providers did not inform them about gynecologic cancer screening recommendations, and about half of the women were not adherent to screening guidelines. Providers’ knowledge (including family physicians and oncologists) was a key factor in determining appropriate risk management strategies for female LS carriers, and those who did not perceive their providers as knowledgeable were less likely to engage in appropriate screening [20].

The factors underlying prophylactic surgical decisions in female LS mutation carriers are not well understood, nor is there sufficient evidence to assess whether women are making informed decisions [18]-[21]. Research is needed to determine the best model to educate women about their risks and options. This study aims to contribute to the evidence base by soliciting the opinions of women affected by LS about their risk-management decisions and experiences.

## Methods

This was an exploratory pilot project designed to investigate the surgical decisions of female LS mutation carriers. Guided by these results, future research will administer a survey to the larger population of women who have made surgical decisions in our jurisdiction. The project was approved by the local Health Research Ethics Authority (HREA).

### Purposive sampling

In Newfoundland and Labrador (NL), Canada, the gynecologic oncology service is located at the Health Sciences Centre (HSC) in the capital city of St. John’s, NL. Women can be referred through their family physicians or other specialists (e.g., oncologists, obstetricians) who note a strong family history of gynecological cancer. Women may also be referred to this speciality service through the Provincial Medical Genetics Program (PMGP), the province-wide genetics service, also located at the HSC in St. John’s.

The PMGP, as well as the gynecological oncology service, maintains a Provincial Cancer Genetics database of all women affected by inherited cancers in the province. Women affected by LS who had undergone prophylactic gynecological surgery within the previous five years were identified through this database. We also searched for female carriers of LS who chose not to have surgery, and identified one who could participate during the recruitment period. Women were purposively sampled to provide a breadth of experience with surgery decisions (e.g., those who lived in rural and urban areas, of different ages, and those for whom varying amounts of time had passed since surgery). These women were contacted by telephone by their physician and asked to call the research team if they were interested in participating. In total, 14 potential participants were approached with ten eventually completing an interview. Time constraints during the data collection period precluded four women from participating. Consent was obtained via post before the interview, and verbal consent at the interview.

### The interviews

Interviews were conducted by telephone between April 2012 and June 2013 by HE and ED. Since most participants lived outside the study area, telephone interviews were chosen as the most convenient by all participants. With permission, interviews were tape-recorded and transcribed verbatim. Interviews lasted from approximately 30 min to 1 hour. A detailed question guide facilitated the tracking of all questions asked during each interview, and women were encouraged to discuss other issues they felt were important. Interviews covered a core set of topics such as family experience of cancer, genetic testing and surgery decisions, impact of the surgery and information needs.

### Data analysis

Qualitative description [22] was used to explore and summarize women’s surgery decisions. This is a form of naturalistic inquiry that makes no *a priori* theoretical or philosophical assumptions about the data. Rather, it seeks to present the data in the language of participants, without aiming to present the data in more theoretical ways. The end result is a comprehensive summary of the event in question [22].

Transcripts were read and re-read several times by HE. Data were then isolated and organized around interview topics; only data pertaining to surgery decisions were utilized for the current analysis. These sections were read and re-read to identify and index emerging categories and themes, which were annotated on the transcripts. No qualitative software was used in the analysis. Inductive subcoding of the data relevant to surgery decisions and information needs was completed using the method of constant comparison [23],[24]. Here, data were compared between and within transcripts to establish analytical categories and themes [23]. This method required a constant shifting back and forth between (and within) transcripts to continuously compare the perceptions and experiences of participants. Discussion with a second investigator (ED) throughout the analysis verified emerging categories and themes until no new themes could be added. Thematic findings were then discussed with the larger research team. No new categories or themes were suggested after team discussion, and data saturation was deemed complete.

## Results

### Participants

Of the 14 women approached for the study, ten eventually completed an interview. The four non-respondents were not different from the included sample in age range, family history of cancer or surgery decision (e.g., all had undergone prophylactic gynecological surgery). Thus we have no reason to believe their exclusion biased the results. Ten participating women ranged from 33–64 years, with a mean age of 49 years. Nearly all had a partner and had adult children. All women were LS mutation carriers, with nine choosing prophylactic surgery. An average of about three and a half years had passed since the time of their surgery, though this was variable (Range: 6 weeks to 8 years). Most women had had prophylactic hysterectomy, as well as oophorectomy (Table 1).

**Table 1:** Demographic and clinical information of interview participants

Participant	Current age	Age at surgery	Mutation	Year of surgery	Type of surgery	Prior cancer?	Use of hormone replacement therapy?
**Sue**	52	48	MSH2	2009	Total abdominal hysterectomy and bilateral salpingo-oophorectomy	Yes, colon	No
**Mavis**	45	41	MSH2	2010	Laproscopic assisted vaginal hysterectomy and bilateral salpingo-oophorectomy	No	Yes
**Delores**	58	55	MLH1	2010	Laproscopic assisted vaginal hysterectomy and bilateral salpingo-oophorectomy	No	Intermittent use
**Cheryl**	52	44	MSH2	2005	Laproscopic assisted vaginal hysterectomy and bilateral salpingo-oophorectomy	No	Yes
**Linda**	33	33	MSH2	2013	Laproscopic assisted vaginal hysterectomy and bilateral salpingo-oophorectomy	No	Yes
**Tonya**	37	Decided against surgery	MSH2	N/A	N/A	No	N/A
**Laura**	64	61	MSH6	2010	Laproscopic assisted vaginal hysterectomy and bilateral salpingo-oophorectomy	Yes, colon and breast	No
**Madonna**	57	56	MSH2	2012	Laparoscopic Right oophorectomy	No	No
**Andrea**	45	44	MSH6	2011	Laproscopic assisted vaginal hysterectomy and bilateral salpingo-oophorectomy	No	No
**Erin**	49	42	MSH2	2006	Total abdominal hysterectomy and bilateral salpingo -oophorectomy	No	No

Study findings are organized around two key themes: 1) prophylactic gynecological surgery decisions, and 2) information provision and needs (Table 2). Most women did not describe their choice of surgery as a ‘decision,’ but something that had to be done. Several interacting factors seemed to underlie this perception, including personal history of cancer and/or a history of abnormal gynecological events (e.g., menstrual issues; ovarian cysts), as well as empathetic knowledge of others’ cancer experiences. These factors created a high level of worry about cancer, and surgery was seen as a way to avoid cancer and lessen worry. Physician recommendation, along with demographic factors, also influenced surgery decisions. Often, a combination of these factors seemed to influence decisions, as revealed in the narratives below.

**Table 2:** Main themes and subthemes arising from interview data

Main themes	Subthemes
1. Factors associated with prophylactic gynecological surgery decisions	Wanting to mitigate cancer worry
	Empathetic knowledge of others’ cancer experiences
	Physician recommendation
	Demographic factors such as age and parity
2. Information provision and needs	Feeling adequately informed prior to surgery
	Needing time to process information prior to surgery
	Information needs, including the sudden onset of surgical-induced menopausal symptoms and the risk and benefits of hormone replacement therapy

### Prophylactic gynecological surgery decisions

#### Mitigating cancer worry

A personal or strong family history with cancer or a history of abnormal gynecological events influenced women’s surgery decisions.

I can make decisions like that pretty quick when it comes to cancer. To cut out anything as long as there is not any cancer there. -Laura, 64 yrs, 2 prior cancers

I was bleeding all the time, my womb was full of fibroids, my ovaries were enlarged…so it probably would have developed into something. -Sue, 52 yrs, prior cancer

With a strong family history of cancer, Linda described the motivation behind her surgery decision simply, “Take that worry off my plate. It has to go.”

#### Empathetic knowledge

Knowledge of the cancer experiences of others seemed to propel some participants in deciding to undergo prophylactic surgery. Linda recalled:

…one of my friends was just diagnosed with ovarian cancer…she’s not doing too good…seeing her and knowing she was a perfectly healthy woman and, all of sudden, she had these big tumors. It happened to her so quick, it could happen to me that quick too. -Linda, 33 yrs, no prior cancer

Similarly, Madonna explained:

And here was my friend going through ovarian cancer; she died a month and a half ago. That kind of put the top on it for me. -Madonna, 57 yrs, no prior cancer

#### Physician recommendation

The advice of physicians also influenced women’s surgical decisions:

He [family doctor] made sure I was getting tested for everything. And the doctors in there [cancer clinic] did mention it, because Mom had had cancer a couple of times, I could have the hysterectomy and at least that way, I’d be safe. –Erin, 49 yrs, no prior cancer

Her strong family history of cancer, coupled with abnormal periods, prompted her physician to recommend prophylactic surgery:

I was having a real lot of trouble with my periods, and a lot of pain…He told me that more than likely, by the time I was 45, I should have the hysterectomy done anyway. So that just helped me along to decide to have it earlier than later.

Sue also discussed her options with her family physician:

So I used to go back to my family doctor, and he said, ‘with your risk of cancer, have you considered having everything taken out?’ And I said, ‘Yes, because of the cancer, and I can’t go on like this. –Sue, 52 yrs, prior cancer

#### Demographic factors – age and childbearing considerations

Mavis explained that her childbearing was complete:

I wanted one child, and that was it. I knew I was done having children. ..I was a little concerned about losing my ovaries and uterus at the time, but I thought it was probably the best thing to do. –Mavis, 45 yrs, no prior cancer

Andrea recalled:

And with me having the gene and talking about hysterectomy, and I said, ‘take it out.’ I don’t have any children, I don’t want any children…that’s the only purpose for it to be there. I said, ‘so I want it taken out.’ –Andrea, 45 years, no prior cancer

The lone participant who decided to engage in intensive screening, rather than prophylactic surgery explained:

It’s a lot to take in. I’m only 37 years old…I’m still young…to go through a surgery and put yourself in early menopause? I don’t know. There’s a lot to think about. I mean, there were other alternatives. –Tonya, 37 yrs, no prior cancer

These narratives suggest that women’s surgical decisions were driven by complex interactions of personal, social and medical factors. No woman described her decision as particularly difficult; all expressed the sense that there was little choice given their personal risk for gynecological cancer. Further, no woman expressed regret about having had surgery. All explained the surgery helped alleviate the fear of developing a gynecological cancer. However, it is not meant to suggest that these decisions were somehow ‘easy’ for women. As Cheryl recalled:

I was in my early 40s, and I’m going to have my uterus, my cervix and ovaries taken out and they’re healthy right now? And I’m not having any gynecological issues at all. That’s major surgery. I’m off work for six weeks. I had a toddler at home that I couldn’t pick up. I go right into surgical menopause. We’re not that long married, and there are issues around sexual response. All those things go through your mind. –Cheryl, 52 yrs, no prior cancer

### Information provision and needs

#### Feeling informed

Most women reported they had received enough information to make informed surgery decisions and were satisfied with the care they received.

I think I was really well prepared going into the surgery…[surgeon] was telling me all the risks and all the benefits. –Linda, 33 yrs, no prior cancer

Similarly, Delores explained:

I had an idea of what I wanted to do if the option was available to me, you know, partial versus full [hysterectomy]. I also did a lot of reading and even looked up the surgery itself, so I knew what to expect. –Delores, 58 yrs, no prior cancer

When asked if she understood her cancer risks, as well as screening and surgery options, Andrea simply said:

Oh yes, she went through all that. I understood what she was saying. –Andrea, 45 yrs, no prior cancer

#### Needing time to process information

Despite feeling they had made informed surgical decisions, women noted the complexity of the information and suggested time was needed to fully process their options.

It was pretty overwhelming at the time…my actual meeting with the genetic counselors when I got my results back is a blur. I don’t remember in any detail what was said to me, but I was given written material. I was able to later read all of that thoroughly and understand it. –Cheryl, 52 yrs, no prior cancer

[genetic counselor] had given me a lot of information, and until you go home and look at it, you don’t really take it all in. …but she’s [gynecologist] been very informative, and she’s been talking to me about it [surgery]. –Tonya, 37, no surgery, no prior cancer

#### Information needs

Study findings reveal gaps in information provided to women who undergo prophylactic gynecological surgery. The potential implications of surgically-induced menopause, as well as the severity of symptoms were mentioned by several women. Some also noted they were unaware of the possibility of hormone replacement therapy.

I didn’t feel I had enough information, no. I think I should have been more informed about the hot flashes and stuff about the sexual drive. –Sue, 52 yrs, prior cancer

When asked if she had discussed surgically-induced menopause with care providers prior to surgery, Erin said:

I don’t think so. I’m not sure if anyone did or not. –Erin, 49 yrs, no prior cancer

Many women also could not recall discussing hormone replacement therapy:

No, not that I can recall. –Madonna, 57 yrs, no prior cancer

No, that was never mentioned. –Laura, 64 yrs, 2 prior cancers

When this subject is bought up to a woman getting a hysterectomy, there needs to be some more information about the hormone replacements that are available…even something that was printed about the best options or what’s out there to be handed out. –Mavis, 45 yrs, no prior cancer

## Discussion

Findings reveal that surgical decisions are complex, driven by interacting demographic, medical and psychosocial variables. Most women recalled no ‘choice’ about surgery, due to their high risk for gynecological cancer and need to reduce levels of cancer worry. Findings support the larger BRCA literature which suggests reducing cancer worry is a key motivator of prophylactic surgical decisions [12],[13]. Women noted other influences on their decisions, including abnormal gynecological screening results, the advice of healthcare providers, and demographic variables. Abnormal gynecological screening results can prompt further risk-management discussions with healthcare providers about surgery, rather than have a negative psychosocial impact on women [18]. No woman in our study reported feeling pressure from providers to choose surgery, and results suggest that regular appointments with clinicians to discuss risk-management options would be well tolerated rather than waiting until an abnormal screening test initiates the discussion.

In line with previous research [13],[18], considerations such as the completeness of childbearing and age also influenced surgery decisions. Findings highlight the importance of timing in the decision to have prophylactic surgery and the need to re-visit screening vs. surgery decisions in the presence of changing personal circumstances. Knowledge of others’ cancer may also affect women’s surgical decisions. Some women explained how seeing friends affected with ovarian cancer contributed to their decision. We have previously described this influence on prenatal screening decisions as ‘empathetic knowledge,’ or subjective knowledge arising from close associations with others [25]. Through empathetic knowledge, women may give meaning to their own cancer risk or decide that the timing is right for surgery. In the experiential knowledge framework, knowledge production is not static and may be revised at any time in response to new experiences and relationships [25],[26]. We suggest this knowledge may be an important influence on cancer risk perception and worry, and can impact risk-management decisions. An exploration of women’s social knowledge may help assess whether there are any misunderstandings or information gaps. d’Agincourt-Canning [27] suggested that genetic counselors probe more deeply their clients’ experiences and corresponding experiential knowledge. She suggested that in addition to collecting family history information, counselors discuss other issues such as care giving experiences or perceptions of cancer’s survivability in order to better understand women’s risk perception. Such discussions may also be valuable in understanding LS carriers’ perceptions of their cancer risks and risk-management options in order to promote informed surgical decision-making.

Most women did feel informed to make surgical decisions; although, some noted that adequate time was needed outside the clinic to process the vast amount of information provided. While most women were satisfied with the information they received, some gaps were noted, such as the severity of surgically-induced menopausal symptoms and the availability of hormone replacement therapy. A minority of women recalled that while they were aware of surgically-induced menopause, they did not appreciate its speed of onset and potential intensity. These findings are in line with recent research [28], which reported nearly 60% of women who had undergone prophylactic oophorectomy would have liked more information about the impact of the surgery on their sex lives. These findings and ours suggest that while many women are well informed about surgically-induced menopause, it is apparent that not all appreciate how quickly menopause can set in following surgery, nor the effect it can have on their sexual relationships. Thus, women should be encouraged to carefully consider the impact surgery can have on their sexuality and be provided with proactive strategies to facilitate sexual adjustment in the event of difficulty [13],[28].

Women should also be given information about the risks and benefits of hormone replacement therapy (HRT), though evidence suggests they may not be given adequate information in this regard [15]. Of course, clinical judgement will be necessary in discussions about HRT. In our sample, some women were in their 50s and 60s at the time of their surgery and their older age could have affected whether discussions were held about HRT. While several women in our study either could not recall being given such information or said they did not receive it, research suggests women at increased risk for ovarian cancer want as much information as possible about their risk-management options, and our findings corroborate these earlier studies [15],[21]. We note that no women in our study suggested she would have chosen differently or that a lack of information somehow impeded her surgery decision. Nonetheless, they did highlight some areas where additional information could be provided, and we hope this is useful information for providers who are caring for women with Lynch syndrome.

Finally, given these information needs and the finding that physician recommendation was a motivating factor for surgery, study findings suggest that awareness of LS cancer risks and management guidelines can be improved among providers, including family physicians and oncologists [19],[20]. This is consistent with other studies observing limitations in providers’ knowledge and management of hereditary cancer syndromes [20],[26]. Like others [19],[20], we suggest that continued efforts to educate providers about screening recommendations for high-risk patients is an important focus for future research.

## Conclusions

This small pilot study revealed motivations behind the prophylactic surgical decisions of women affected with LS, as well as their perceptions about pre-surgical information provision and needs. However, some study limitations are noted. The sample size is very small, and the study was conducted in a comprehensive cancer service; findings may not generalize to patients seen outside such centres. Further, we could only recruit one patient who declined surgery and opted for surveillance instead, and as such, findings cannot be generalized to women declining surgery. Data rely on patient recollection of pre and post-surgical discussions with providers. We did not collect data on the content of these discussions, and the data may suffer from recall bias. Despite these limitations, study findings are in line with the growing literature on LS carriers and highlight areas for discussion in risk-management counseling and implications for information provision regarding prophylactic surgery.
